# Generalized analysis of dynamic pull-in for singular magMEMS and MEMS oscillators

**DOI:** 10.1038/s41598-025-09515-9

**Published:** 2025-07-03

**Authors:** Piotr Skrzypacz, Grant Ellis, Bartosz Pruchnik, Piotr Putek

**Affiliations:** 1 Department of Mathematics, School of Sciences and Humanities, Nazarbayev University, Kabanbay Batyr 53, 010000 Astana, Kazakhstan; 2 Faculty of Electronics, Photonics and Microsystems, Wrocław University of Science and Technology, ul. Janiszewskiego 11/17, 50-372 Wrocław, Poland

**Keywords:** Singular oscillators, Dynamic pull-in, MEMS, MagMEMS, Periodic solutions, Electrical and electronic engineering, Mechanical engineering

## Abstract

The exact pull-in threshold for single-degree-of-freedom actuator models arising in the design of micro-electro-mechanical systems (MEMS) is derived analytically by studying exact solutions to trinomial equations of arbitrary degree. In the case of magnetic actuation (magMEMS), the exact pull-in threshold is expressed in terms of the Lambert W function, while in the case of actuation inversely proportional to the positive power of distance, the closed form of the dynamic pull-in threshold is expressed in terms of infinite series or Gaussian hypergeometric functions. Periodic and pull-in solutions for various excitation parameters and actuation exponents are numerically illustrated.

## Introduction

Advances in microfabrication technology have led to remarkable progress in the design and manufacture of micro-electro-mechanical systems (MEMS), where both component dimensions and actuation are in the micro-meter range. Unlike traditional industrial methods for producing mechanical components, surface micromachining and bulk micromachining are employed in the fabrication of the semiconductor elements within an electromechanical integrated system. Due to the increasing refinement of semiconductor manufacturing processes, many highly advanced microstructures are now available, driving innovation in areas such as telecommunications, radar systems, and mobile devices. Consequently, the performance of devices has been significantly optimized in terms of faster response times, reduced power consumption, and compatibility with standard integrated circuit processes^1–4^. In this work, the focus is on the investigation of the dynamics of two key classes of MEMS resonators, such as singular magnetic MEMS (magMEMS) and electrostatic MEMS oscillators.

Pull-in instability of MEMS devices generally is a common but undesirable phenomenon that remains an active area of research due to its direct impact on the performance, profitability, and reliability of microelectromechanical systems, see, e.g.,^5–8^ and references therein. In particular, careful tuning of electrical load parameters during the design and prototyping stages is essential to prevent pull-in, which can lead to structural collapse and device failure^16^. From this perspective, pull-in analysis, typically classified as static or dynamic, is a critical component in the design of MEMS resonators^10,11^. In fact, this phenomenon attracts considerable attention from both researchers and practitioners for several reasons. First, the operational range of MEMS actuators is often limited by pull-in instability, and extending this range requires a precise understanding of the device stability limits. In addition, pull-in analysis offers a valuable and generalized characterization of electrostatic and magnetic actuators. For example, by comparing theoretical predictions with experimental measurements of pull-in parameters, key properties such as the equivalent stiffness of microstructures and various material characteristics can be inferred^2^.

In this context, dynamic pull-in plays a critical role in nanometrology and nanotechnology. For example, spontaneous adhesion of closely spaced nanostructures due to electrostatic forces may occur during nanomanipulation^22^. Pull-in is typically undesirable in scanning probe microscopy (SPM) techniques, as it leads to non-uniform adhesion of the probe to the substrate, primarily caused by van der Waals forces. However, it can be harnessed for measurement purposes in electrostatic or magnetic force microscopy (EFM and MFM, respectively). From a physical perspective, a dynamic pull-in refers to the collapse of an elastic structure on a substrate as a result of the interaction between kinetic energy and potential energy^1^. This effect can also be interpreted as an escape from the potential well of microelectromechanical systems according to Elata and Bamberger^12^ and Sedighi^13^. Accordingly, both numerical and analytical approaches are employed to examine the pull-in instability as a fundamental constraint in electrostatic, electromagnetic, and magnetic MEMS devices. The authors in^9,14,15,17,18^ have explored the dynamics of damped nonlinear microbeam models using numerical methods to approximate the pull-in threshold. The second group of methods involves the analytical solution of the nonlinear undamped spring-mass equation with initial conditions driven by various types of forcing terms, such as the electrostatic Coulomb force, Lorentz force, or van der Waals attraction and repulsion, see, e.g.,^4,7,9,19^.

More specifically, for the mass-spring system, the dynamic pull-in refers to the collapse of the moving structure due to the interchange between kinetic and potential energies. Mathematical analysis of single-degree-of-freedom magMEMS/MEMS oscillators has been presented in our previous studies^4,20,21^. While the pull-in phenomenon in electrostatically (Coulomb force) and magnetostatically (Lorentz force) actuated MEMS is fairly well understood, devices actuated by other types of forces require further investigation. Here, MagMEMS/MEMS oscillators subjected to generalized actuation mechanisms are considered. In nanostructures, quantum mechanical effects, such as Casimir forces, which are inversely proportional to the fourth power of the distance, become significant^23^. In submicrometer MEMS devices, other adhesion effects, including capillary, electrostatic, and van der Waals forces, may also play a role^24^. In^25^, magnet-magnet interactions exhibit attraction forces that scale with the inverse of the distance raised to the eighth power.

In this study, our objective is to precisely determine the dynamic pull-in threshold in MEMS devices, specifically, the minimum voltage or current required to trigger pull-in. To this end, we utilized the Lambert W function to express the exact pull-in threshold for magnetic actuation (magMEMS). Additionally, we derive closed-form expressions for the dynamic pull-in threshold when the actuation is inversely proportional to the positive power of the distance, representing these thresholds through infinite series or Gaussian hypergeometric functions. We also present numerically computed periodic and pull-in solutions for various excitation parameters and actuation exponents.

The paper is organized as follows. The single-degree-of-freedom model for magMEMS/MEMS oscillators is introduced in “Mathematical model of magMEMS/MEMS oscillators”. In “Dynamic pull-in threshold”, we derive the generalized expressions for the dynamic pull-in threshold and the maximum amplitude. Applications are discussed in “Applications”, including an analysis of the physical parameters necessary for pull-in in two magMEMS devices. Finally, the findings are summarized in “Conclusions”.

## Mathematical model of magMEMS/MEMS oscillators

In the following, we describe the basic principles in systems based on the actuation resulting from the force of attraction between the fixed and the flexible part. We assume that the actuating force is inversely proportional to a certain power, *m*, of the distance between them, i.e.,

1 $$\begin{aligned} F_A=\frac{\kappa }{(b-\tilde{x})^m},\quad m\geqslant 0. \end{aligned}$$

Here *b* is the maximum distance between the flexible and fixed parts and the parameter $\kappa$ is related to the physical MEMS design. The attractive force in Eq. (1) can be generated by magnetic or electric fields. For example, the parameter $\kappa$ for the magMEMS with current carrying wires of length *L* and based on magnetic actuation ($m=1$) is given by^21^

$$\begin{aligned} \kappa =\frac{\mu _0i_1i_2L}{2\pi }, \end{aligned}$$

where $i_1, i_2$ denote the electrical current and $\mu _0$ is the electrical permeability of the vacuum. The parameter $\kappa$ for the MEMS representing the corresponding parallel-plate capacitor and based on electrostatic actuation ($m=2$) is given by^1^

$$\begin{aligned} \kappa =\frac{\varepsilon _0AV_{DC}^2}{2}, \end{aligned}$$

where *A* is the area of the capacitor cover, $V_{DC}$ denotes the DC voltage and $\varepsilon _0$ represents the electrical permittivity of the vacuum.

As mentioned earlier, magnet-magnet interactions have attractive forces with exponents up to $m=8$. The dynamic single degree of freedom differential equation describing the motion of a flexible part such as a cantilever tip (considered as a point of mass *M*) can be derived using Newton’s second law of motion as

$$\begin{aligned} M\dfrac{d^2\widetilde{x}}{d\tilde{t}^2}=F_R+F_A, \end{aligned}$$

where $F_R=-k_s\widetilde{x}$ is the restoring force of a linear spring element, and $F_A$ is the attraction force by Eq. (1). Here, $k_s$ denotes the spring constant. In the model considered, dissipation forces, such as damping, are omitted.

As usual, let us introduce *x*, *t* as the dimensionless distance and time, respectively, as follows

2 $$\begin{aligned} x=\frac{\widetilde{x}}{b}\quad \text {and}\quad t=\widetilde{t}\,\sqrt{\frac{k_s}{M}}. \end{aligned}$$

Next, we define the excitation parameter as

3 $$\begin{aligned} K=\frac{\kappa }{k_s\,b^{m+1}}. \end{aligned}$$

Then, the dynamic differential equation can be rewritten in the dimensionless form as

4 $$\begin{aligned} \ddot{x}+x=\frac{K}{(1-x)^m}, \qquad x<1, \end{aligned}$$

where $\ddot{x}$ denotes the second derivative with respect to time. Finally, to solve a problem (4), we have to prescribe zero initial conditions $x(0)=\dot{x}(0)=0$, and assume that $K>0$.

## Dynamic pull-in threshold

**Fig. 1:**
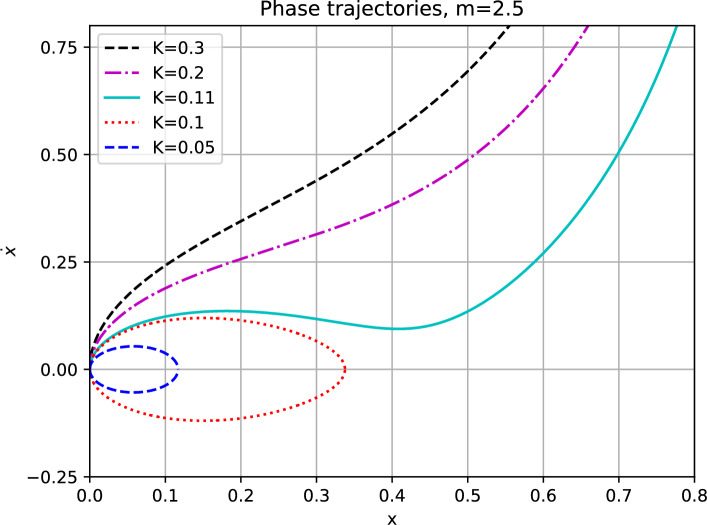
The zero-initial value problem has periodic solution for $m>0$ if the orbits are closed.

Multiplying Eq. (4) by $\dot{x}$ and integrating with respect to time, for $m>0$, yields the conservation of energy in the following form

$$\begin{aligned} \mathcal {E}_{K,m}(t)= {\left\{ \begin{array}{ll} \dfrac{1}{2}(\dot{x}(t))^2+\dfrac{1}{2}x^{2}(t) +K\ln {(1-x(t))}, & \quad m=1,\\ \dfrac{1}{2}(\dot{x}(t))^2+\dfrac{1}{2}x^{2}(t)-\dfrac{K}{(m-1)(1-x(t))^{m-1}}+\dfrac{K}{m-1}, & \quad m\ne 1, \end{array}\right. } \end{aligned}$$

which $\mathcal {E}_{K,m}(t)\equiv 0$ due to zero initial conditions. As a result, we obtain

5 $$\begin{aligned} (\dot{x}(t))^2=f_{K,m}\bigl (x(t)\bigr ), \end{aligned}$$

where

6 $$\begin{aligned} f_{K,m}(s)= {\left\{ \begin{array}{ll} -s^{2} -2K\ln {(1-s)}, & \quad m=1,\\ -s^{2}+\dfrac{2K-2K(1-s)^{m-1}}{(m-1)(1-s)^{m-1}}, & \quad m\ne 1, \end{array}\right. } \end{aligned}$$

from which we immediately infer that for $m\geqslant 1$ the solution *x*(*t*) is non-negative for all $t\geqslant 0$. The pull-in analysis is based on studying phase diagrams for several values of the excitation parameter *K*, see Fig. 1. For $m= 2.5$, the dynamic pull-in threshold is $K^* = 0.1047652971\ldots$.

The closed orbits in phase diagrams represent periodic solutions that exist for $0<K<K^*$ where $K^*$ is called the dynamic pull-in threshold. The orbits are closed when the function $f_{K,m}(s)$ of Eq. (6) has a root in (0, 1). Also, notice that the smallest positive root of $f_{K,m}(s)$ in (0, 1) represents the maximal deflection of the oscillating part in magMEMS/MEMS.

**Fig. 2:**
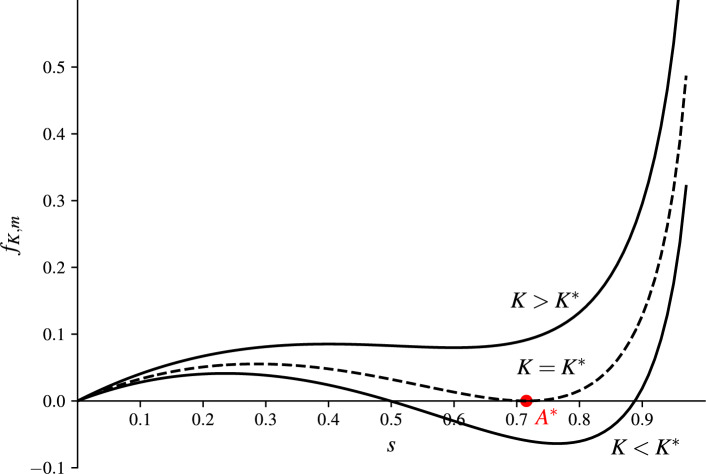
The zero-initial value problem has periodic solution for $m>0$ if $f_{K,m}(s)$ has a root in (0, 1).

For a given $m>0$, the pull-in occurs if $K>K^*$. This is the case when $f_{K,m}(s)$ has no roots in (0, 1). If $K=K^*$, then the function $f_{K,m}(s)$ has a double root $A^*$ in (0, 1), see Fig. 2. For this case if the pair $(K^*, A^*)$ satisfies the following algebraic system

7 $$\begin{aligned} f_{K^*,m}(A^*)=0\quad \text {and}\quad \frac{\partial f_{K^*,m}}{\partial s}(A^*)=0. \end{aligned}$$

The following theorem determines the exact value of the dynamic pull-in threshold for magMEMS ($m=1$).

### Theorem 1

*Let* $m=1$. *Then, the dynamic pull-in threshold* $K^*$ *for the zero-initial value problem* (4) *is given by*

$$\begin{aligned} K^*=-\left( 2W_{-1}\!\left( -e^{-1/2}/2\right) +1\right) W^{-2}_{-1}\!\left( -e^{-1/2}/2\right) /4\doteq 0.2036321888. \end{aligned}$$

*where* $W_{-1}:[-1/e,0)\rightarrow [-1,-\infty )$ *denotes the second branch of the LambertW function which is defined as a solution* $y(x)\leqslant -1$ *of the transcendental equation* $ye^{y}=x$ for $x\in [-1/e,0)$^26^.

### Proof

If $m=1$, then the nonlinear algebraic system by Eq. (7) becomes

$$\begin{aligned} -(A^*)^2-2K^*\ln {(1-A^*)}=0\quad \text {and}\quad -2A^*+2K^*/(1-A^*)=0, \end{aligned}$$

from which we infer $K^*=A^*-(A^*)^2$ and $-A^*-2(1-A^*)\ln {(1-A^*)}=0$. The latter equation can be rewritten as $-\frac{1-2t^*}{2}e^{-\frac{1-2t^*}{2}}=-\frac{1}{2}e^{-1/2}$ where $t^*=\ln {(1-A^*)}$. Now, recalling the definition of the LambertW function, we can find the root of this equation using $-(1-2t^*)/2=W_{-1}\left( -e^{-1/2}/2\right)$. Note that the solution $W(-e^{-1/2}/2)=-1/2$ leads to $A^*=0$. Therefore, $1-2t^*=-2W_{-1}\left( -e^{-1/2}/2\right)$ and $A^*=1-\exp \left( t^*\right)$. Consequently,

8 $$\begin{aligned} \begin{aligned} A^*&=1-\exp \left( W_{-1}\left( -e^{-1/2}/2\right) +1/2\right) \\&=1+W_{-1}^{-1}\left( -e^{-1/2}/2\right) /2\\&\doteq 0.715331863, \end{aligned} \end{aligned}$$

and the assertion follows from $K^*=A^*(1-A^*)$ mentioned above. $\square$

Note that in^21^, the numerical value $K^*=0.2036321888$ was previously derived as a solution to the transcendental equation

$$\begin{aligned} (1+\sqrt{1-4K^*})^2/4+2K^*\ln {(1/2-\sqrt{1-4K^*}/2)}=0 \end{aligned}$$

which follows from the algebraic system by Eq. (7). The following theorem gives the exact value of the dynamic pull-in threshold for MEMS ($m\in {{\mathbb {N}}}$ and $m\ne 1$).

### Theorem 2

*Let* $m\geqslant 2$ *be an integer*. *Then, the dynamic pull-in threshold* $K^*$ *for the zero-initial value problem* (4) *is given by*

9 $$\begin{aligned} K^*=\frac{m-1}{2}\left( 1-\frac{m-1}{m+1}G_m\right) \left( G_m-1\right) , \end{aligned}$$

*where* $G_m$ *is defined in terms of the Gauss hypergeometric function* $\phantom {a}_m F_{m-1}$^26^ *as follows*

10 $$\begin{aligned} G_m= \phantom {a}_m F_{m-1}\left( \begin{array}{lll} \frac{1}{m} & ,\ldots , & \frac{m}{m} \\ \frac{2}{m-1} & ,\ldots , & \frac{m}{m-1} \end{array}\Biggl \arrowvert 2\left( \dfrac{m}{m+1}\right) ^m\right) . \end{aligned}$$

### Proof

If $m\ne 1$, then the algebraic system by Eq. (7) becomes

11 $$\begin{aligned} \begin{aligned} (m-1)(A^*)^2(1-A^*)^{m-1}+2K^*(1-A^*)^{m-1}-2K^*&=0,\\ (m+1)(A^*)^2-2A^*+2K^*&=0, \end{aligned} \end{aligned}$$

from which we infer

12 $$\begin{aligned} (t^*)^m-\frac{m+1}{2}t^*+\frac{m-1}{2}=0, \end{aligned}$$

where $t^*=1-A^*$. Using $t^*=\left( \frac{m+1}{2}\right) ^{\frac{1}{m-1}}z$, Eq. (12) can be transformed to the trinomial equation

$$\begin{aligned} z^m-z+a=0\quad \text {with}\quad a=\frac{m-1}{2}\left( \frac{m+1}{2}\right) ^{\frac{m}{1-m}} \end{aligned}$$

whose root is given by^27^

$$\begin{aligned} z_m=a\, \phantom {a}_m F_{m-1}\left( \begin{array}{lll} \frac{1}{m} & ,\ldots , & \frac{m}{m} \\ \frac{2}{m-1} & ,\ldots , & \frac{m}{m-1} \end{array}\Biggl \arrowvert m\left( \dfrac{ma}{m-1}\right) ^{m-1}\right) , \end{aligned}$$

i.e.,

13 $$\begin{aligned} z_m=\frac{m-1}{2}\left( \frac{m+1}{2}\right) ^{\frac{m}{1-m}} \phantom {a}_m F_{m-1}\left( \begin{array}{lll} \frac{1}{m} & ,\ldots , & \frac{m}{m} \\ \frac{2}{m-1} & ,\ldots , & \frac{m}{m-1} \end{array}\Biggl \arrowvert 2\left( \dfrac{m}{m+1}\right) ^m\right) . \end{aligned}$$

It follows from the second equation in the algebraic system by Eq. (11) that

$$\begin{aligned} K^*=-(m+1)(A^*)^2/2+A^*. \end{aligned}$$

Using

14 $$\begin{aligned} A^*=1-t^*=1-z_m\left( \frac{m+1}{2}\right) ^{\frac{1}{m-1}} \end{aligned}$$

and performing some additional algebraic transformations we conclude the assertion. $\square$

### Remark 3

If the restoring force is non-linear, then the algebraic system of Eq. (7) must be modified. In the case of the graphene MEMS oscillator, the linear term *x* in Eq. (4) will be replaced by the non-linear term $x-\alpha |x|x$^28,29^, where $\alpha >0$ corresponds to the graphene material parameter. Consequently, the resulting algebraic equation will also contain the non-negative parameter $\alpha >0$. Techniques for deriving the exact and approximate dynamic pull-in threshold for the general case of $m>0$ will be analyzed in our future research.

**Fig. 3:**
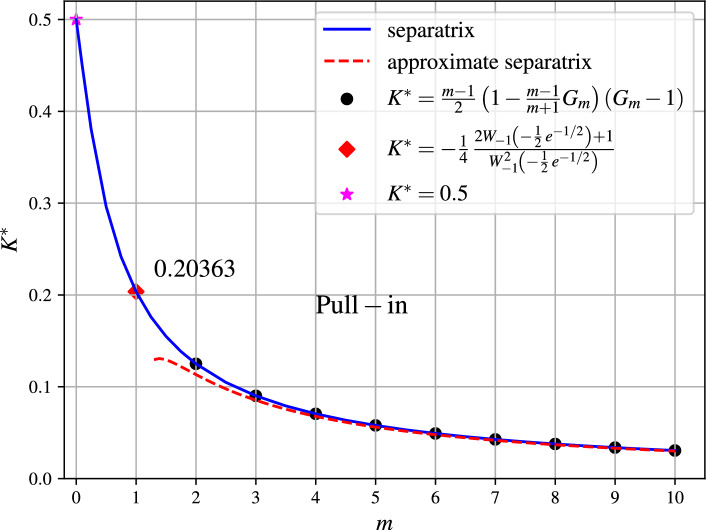
Operational diagram for magMEMS/MEMS. The pairs (*m*, *K*) corresponding to points above the separatrix lead to the pull-in.

In the general case of MEMS ($m>1$), we have the following theorem.

### Theorem 4

*Let* $$m>1$$.

**Fig. 4:**
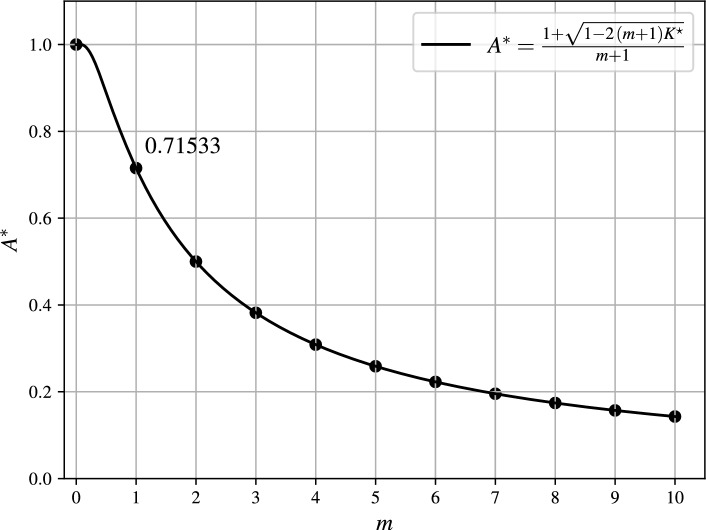
Maximum deflection of oscillating part.

*Then, the dynamic pull-in threshold* $K^*$ *for the zero-initial value problem* (4) *is given by*

15 $$\begin{aligned} K^*=\frac{1}{2}\left( \frac{2}{m+1}-S_m\right) (m+1)S_m, \end{aligned}$$

*where*

16 $$\begin{aligned} S_m=\sum \limits _{j=1}^\infty \frac{\Gamma (mj+1)}{\Gamma (j)\Gamma (mj-j+2)j}\left( \frac{m-1}{m+1}\right) ^{mj+1-j}\left( \frac{2}{m+1}\right) ^j. \end{aligned}$$

*Furthermore, if* $m=0$, *then* $K^*=1/2$.

### Proof

Let $m>1$. Substituting $t^*=\frac{m-1}{m+1}y$, Eq. (12) can be transformed to the trinomial equation

$$\begin{aligned} wy^m-y+1=0\quad \text {with}\quad w=\frac{2}{m-1}\left( \frac{m-1}{m+1}\right) ^m. \end{aligned}$$

Its root can be represented in the form of infinite series^30^

$$\begin{aligned} y=1+\sum \limits _{j=1}^\infty \frac{\Gamma (mj+1)}{\Gamma (j)\Gamma (mj-j+2)} \frac{w^j}{j}. \end{aligned}$$

The assertion follows from

$$\begin{aligned} A^*=1-t^*=1-\frac{m-1}{m+1}y \end{aligned}$$

and

$$\begin{aligned} K^*=A^*-\frac{m+1}{2}(A^*)^2. \end{aligned}$$

If $m=0$, then the zero-initial value problem for Eq. (4) always has a periodic solution $x(t)=K-K\cos {(t)}=2K\sin ^2{(t/2)}$. However, $\max \limits _{t\geqslant 0} x(t)\leqslant 1$ due to the physical constraint $x<1$, and so $A^*=1$. Therefore, $A^*=\max \limits _{t\geqslant 0} x(t)=2K^*=1$ and consequently $K^*=1/2$. $\square$

The effect of *m* on the dynamic pull-in threshold is illustrated in Fig. 3. The approximate separatrix shown in Fig. 3 is defined by the 10-term partial sum of $S_m$ from Eq. (16). The maximum deflection of the oscillating part is specified by $A^*$ in the following lemma.

### Lemma 5

*Let* $m\geqslant 0$. *Then, the maximum deflection of the oscillating part is given by*

17 $$\begin{aligned} A^*=\frac{1+\sqrt{1-2(m+1)K^*}}{m+1}. \end{aligned}$$

*Additionally, it holds for integer* $m\geqslant 2$ *that*

$$\begin{aligned} A^*=1-\frac{m-1}{m+1}G_m \end{aligned}$$

*where* $G_m$ *is defined by Eq*. (10).

### Proof

From the second equation in the algebraic system by Eq. (7) it can be deduced that for $m>0$ the quadratic equation $(m+1)(A^*)^2-2A^*+2K^*=0$ is satisfied. Its larger root is $A^*$ given by Eq. (17). If $m=0$, then $K^*=1/2$ and $A^*=1$ as stated in the proof of Theorem 4. Therefore, Eq. (17) is also valid for $m=0$. If $m\geqslant 2$ is an integer, then the identity $A^*=1-\frac{m-1}{m+1}G_m$ follows from Eq. (14). $\square$

**Fig. 5:**
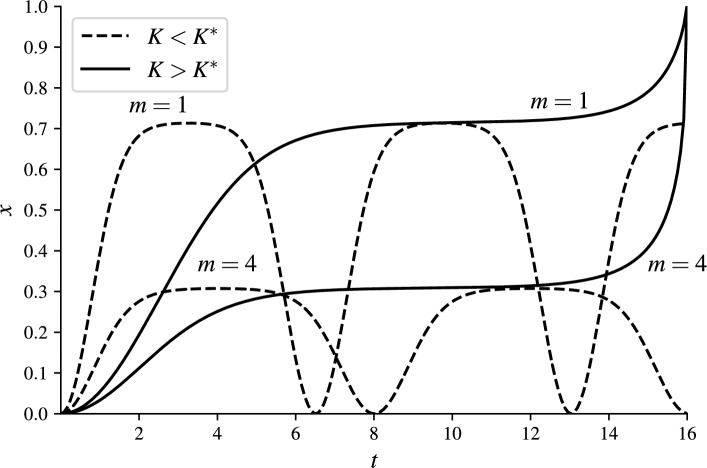
Periodic and pull-in solutions for excitation parameters $K=(1-\delta )K^*$ and $K=(1+\delta )K^*$ with $\delta =1e-5$, respectively.

Note that the series by Eq. (15) from Theorem 4 converges very slowly for values *m* close to 1. Therefore, it is preferable to compute $K^*$ as the solution to the transcendental equation

18 $$\begin{aligned} \begin{aligned}&\frac{m-1}{(m+1)^{m+1}}\left( 1+\sqrt{1-2(m+1)K^*} \right) ^2\left( m-\sqrt{1-2(m+1)K^*}\right) ^{m-1}\\&\quad +2K^*\left( \frac{m-\sqrt{1-2(m+1)K^*}}{m+1}\right) ^{m-1}-2K^*=0, \end{aligned} \end{aligned}$$

which is obtained by inserting $A^*$ by Eq. (17) into the first equation of the algebraic system by Eq. (11). Eq. (18) is also used to calculate $K^*$ for $m\in (0,1)$. The maximum deflection $A^*$ is shown in Fig. 4. It can be observed that the maximum deflection $A^*$ tends to zero as the exponent *m* increases.

### Remark 6

Conventional approaches, including the variational iteration method and the homotopy perturbation method, have been shown to provide approximate pull-in thresholds with a relatively high degree of accuracy^31,32^. For example, J.-H. He used the variational iteration method in^31^ to derive the approximate pull-in threshold for the magMEMS oscillator ($m=1$). The resulting approximation $\tilde{K}^*=0.20498$ of $K^*$ has a percentage inaccuracy of less than $1\%$. Alternative approximations can be obtained from Eq. (18) using iterative solution methods; see^16^. For $m=2.5$, one iteration of the ancient Chinese algorithm^33^ yields the approximation $K^*\approx 0.1049157613K$, with a relative error of $0.14\%$, using the initial iterates $K_1=0.1$ and $K_2=0.12$. The approximate dynamic pull-in thresholds for the general case of $m\ne 1$ deserve separate investigation in our future research.

## Applications

The oscillatory and pull-in solutions for the case of magMEMS ($m=1$) and MEMS ($m=4$) are illustrated in Fig. 5 for the choice of excitation parameter $K=(1\pm \delta )K^*$ with $\delta =1e\text {--}5$. If the excitation parameter *K* exceeds the dynamic pull-in treshold $K^*$, the solution *x*(*t*) reaches at the finite time $t_p$ the level $x=1$. If $K>K^*$, the pull-in time can be computed by integrating Eq. (5) as follows

19 $$\begin{aligned} t_p(K,m)=\int \limits _0^1 \frac{ds}{\sqrt{f_{K,m}(s)}}. \end{aligned}$$

**Fig. 6:**
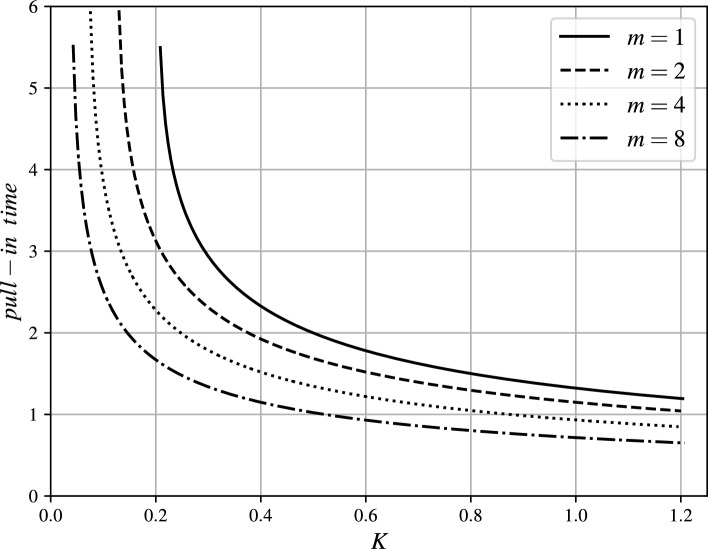
Pull-in time $t_p$ vs. excitation parameter *K* for several values of exponent $m\geqslant 1$.

**Fig. 7:**
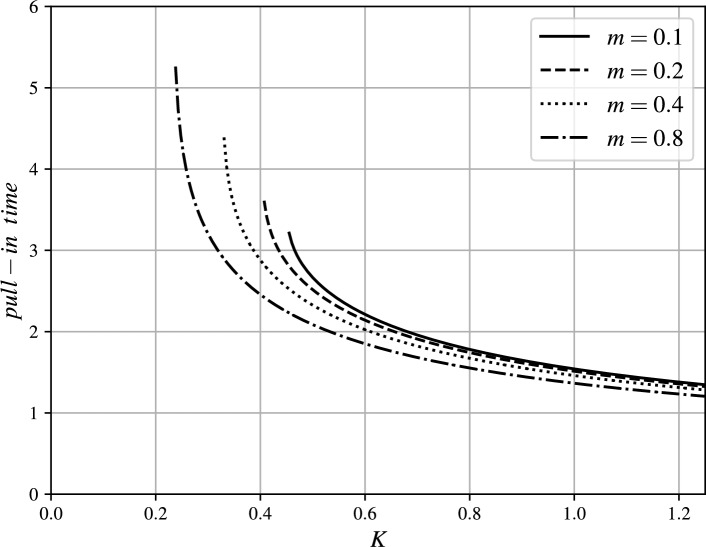
Pull-in time $t_p$ vs. excitation parameter *K* for several values of exponent $0<m<1$.

The trends of $t_p$ vs. *K* are illustrated in Figs. 6 and 7 for several values of $m\geqslant 1$ and $0<m<1$, respectively. The actual pull-in times ($\tilde{t}_p$) for a real application can be determined using the transformation by Eq. (2).

**Fig. 8:**
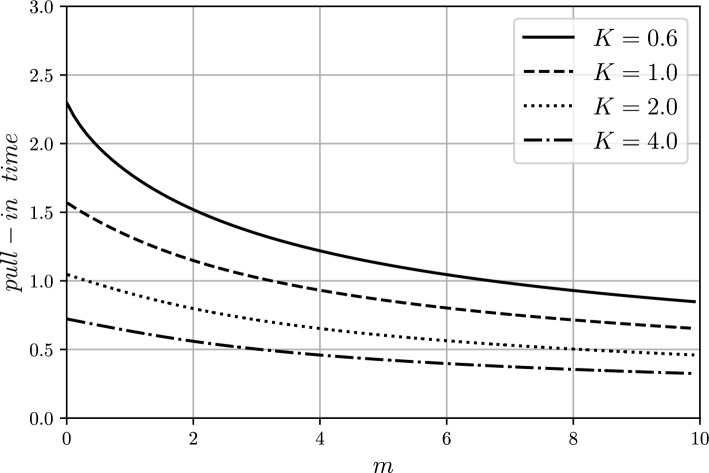
Pull-in time $t_p$ vs. exponent $m>0$ for several values of excitation parameter $K>0$.

For the fixed value of *K*, it holds true that $t_p(K,m_1)<t_p(K,m_2)$ for $m_1>m_2$ due to the fact that the attraction force increases with increasing exponent *m*. This effect of exponent increasing $m>0$ on the pull-in time can be observed in Figs. 6, 7 and 8.

**Fig. 9:**
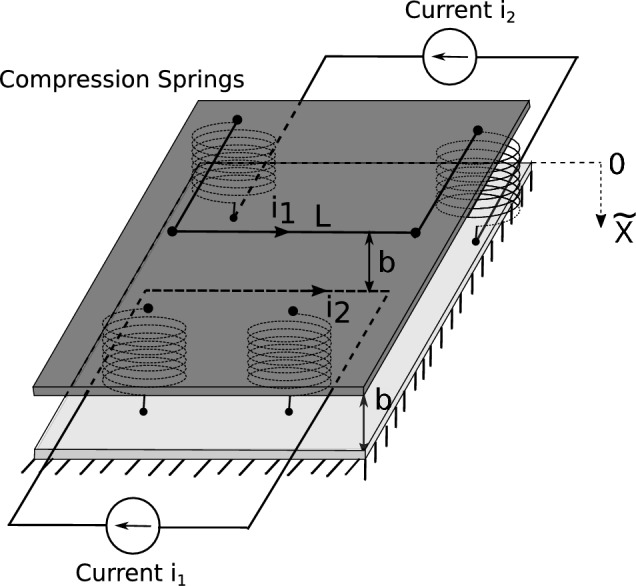
magMEMS with current-carrying filament.

**Fig. 10:**
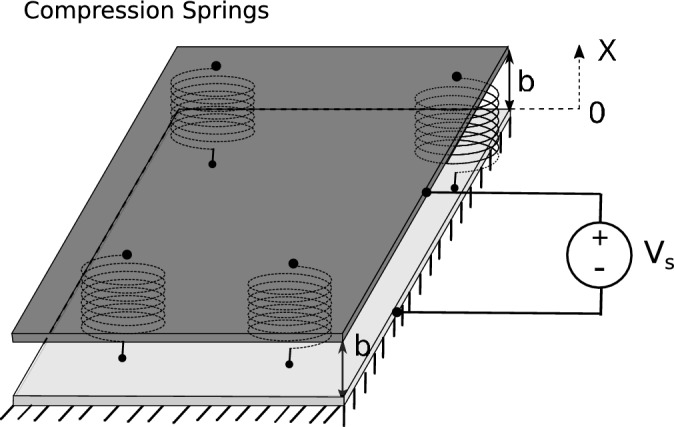
MEMS parallel plate capacitor.

Figures 9 and 10 show possible applications, which are treated here more as academic benchmarks, but contain the main concept of the real devices. The first is an example of magnetic or magMEMS for $m = 1$, while the second corresponds to conventional electric field MEMS for $m = 2$. Obviously, in practice, the production of MEMS devices would require special manufacturing processes. From a technical point of view, MEMS spring constants as low as 0.1 N/m have been fabricated, see for example,^34^. The fabrication of spring constants as low as 0.02128 N/m has also been reported in^35^. The existence of a material called Permalloy that can be used for MEMS fabrication with high relative permeability $\mu _r = 8500$ has been reported in^36^. MagMEMS and electric field MEMS pull-in can be achieved for $K^* \approx 0.2036$. This occurs for the following parameters for magMEMS^21^

$$\begin{aligned} K=\frac{L i_{1} i_{2} \mu _{0} \mu _{r}}{2 \, \pi b^{2} k_s } \end{aligned}$$

where $i_1 = i_2 = 38$ mA, total length of the wire, $L = 20$ mm, spacing $b = 0.1$ mm, spring constant $k_s = 0.1$ N/m, and $\mu _r = 350$ while the parameter for MEMS^4^

$$\begin{aligned} K = \frac{A V_{ DC }^{2} \varepsilon _{0}}{2 \, b^{3} k_s } \end{aligned}$$

is assumed at $V_{DC} = 43$ volts, plate-plate spacing $b = 0.1$ mm, spring constant $k_s = 0.1$ N/m, and plate area of $5\, \text {mm} \times 5\, \text {mm} = 25 \,\text {mm}^2$.

## Conclusions

The exact threshold for dynamic pull-in occurring in models of singular magMEMS/MEMS oscillators subject to a generalised actuation force inversely proportional to a power of the distance between the fixed and flexible parts has been derived in closed form. The results of this study are summarised in Table 1, which presents the effects of varying system parameters, analysed both analytically and numerically.

**Table 1:** Thresholds for dynamic pull-in and maximal deflections of oscillating parts.

m	$K^*$	$A^*$
1	$\displaystyle -\frac{2W_{-1}\! \left( -e^{-1/2}/2\right) +1}{4W^{2}_{-1}\!\left( -e^{-1/2}/2\right) }=0.20363$	$1+W_{-1}^{-1}\left( -e^{-1/2}/2\right) /2=0.71533$
2	0.125	0.5
3	$\displaystyle (-11+5\sqrt{5})/2=0.09017$	$(3-\sqrt{5})/2=0.38197$
4	$\displaystyle \frac{-5\gamma _1\sqrt{113}-8\gamma _1^2+68\sqrt{113}-83\gamma _1+700}{4\gamma _1^2}=0.07052$	$(-\gamma _1/2+4/\gamma _1+4)/3=0.30859$
	$$\gamma _1=(190+18\sqrt{113})^{1/3}$$	
5	$2(1-4\gamma _2/6)(\gamma _2-1)=0.05791$	$1-4\gamma _2/6=0.25873$
	$$\gamma _2=\,\phantom {a}_5 F_4\left( \begin{array}{lll} 1/5 & ,\ldots , & 1 \\ 2/4 & ,\ldots , & 5/4 \end{array}\Biggl \arrowvert 2\left( \frac{5}{6}\right) ^5\right)$$	
6	$5(1-5\gamma _3/7)(\gamma _3-1)/2=0.04912$	$1-5\gamma _3/7=0.22269$
	$$\gamma _3=\,\phantom {a}_6 F_5\left( \begin{array}{lll} 1/6 & ,\ldots , & 1 \\ 2/5 & ,\ldots , & 6/5 \end{array}\Biggl \arrowvert 2\left( \frac{6}{7}\right) ^6\right)$$	

Furthermore, the derived results for periodic and pull-in solutions generalise those previously given in^4,21^. The availability of such closed-form solutions can facilitate the development of single-degree-of-freedom models of magnetically or electrostatically actuated MEMS to enable innovative designs of magMEMS/MEMS devices. For example, the use of a space-mapping algorithm^37^ for optimisation purposes - with an available analytical formula for a coarse model and finite element simulations for a refined model - can significantly accelerate the design and optimisation process of new MEMS devices.

Finally, while future investigations should include experimental studies to verify the theoretical results obtained in this study, even in this form they provide valuable insights into the behaviour of these systems and may inspire further research in this area.

## Data Availability

All data generated or analysed during this study are included in this published article.
